# Comparison of Clinical Outcomes Between Robot-Assisted Esophagectomy With Total Mesoesophageal Excision and Conventional Minimally Invasive Esophagectomy for Esophageal Cancer

**DOI:** 10.1245/s10434-025-18876-4

**Published:** 2026-01-09

**Authors:** Yu Huang, Chi Zhang, Bowen Zhao, Peiyuan Mei, Zuhan Geng, Kuo Li, Quanfu Huang, Lin Zhou, Liqiang Xu, Zaixing Cheng, Yongde Liao

**Affiliations:** 1 Department of Thoracic Surgery, Tongji Medical College Union Hospital, Huazhong University of Science and Technology, Wuhan, Hubei China; 2 Department of Histology and Embryology, School of Basic Medicine, Tongji Medical College, Huazhong University of Science and Technology, Wuhan, Hubei China; 3 Department of Cardiothoracic Surgery, Taihe Hospital, Hubei University of Medicine, Shiyan, Hubei China

**Keywords:** Mesoesophagus, RAMIE, Esophagectomy, Thoracoscopy, Clinical outcomes

## Abstract

**Background:**

This study aimed to evaluate the clinical value of robot-assisted surgery combined with the total mesoesophageal excision (TME) for resectable esophageal cancer and to compare its advantages over conventional minimally invasive esophagectomy (MIE) and non-mesoesophageal esophagectomy.

**Methods:**

The study retrospectively analyzed data from 159 patients who underwent McKeown esophagectomy at 2 provincial tertiary hospitals (January 2019–March 2025). The patients were stratified into 4 groups based on surgical approach, including robot-assisted total mesoesophageal esophagectomy (RATME, *n* = 38), robot-assisted conventional minimally invasive esophagectomy (RAMIE, *n* = 37), video-assisted thoracoscopic total mesoesophageal esophagectomy (VATME, *n* = 42), and video-assisted minimally invasive esophagectomy (VAMIE, *n* = 42). The analysis compared baseline characteristics, perioperative data, and survival outcomes among groups.

**Results:**

The RATME group had a significantly longer operative time than the other groups (*P* < 0.01). However, it demonstrated significant reductions in intraoperative blood loss and thoracic drainage volume within the first 48 h postoperatively (*P* < 0.05), together with a shorter postoperative hospital stay. Compared with the non-mesoesophageal group, the mesoesophageal group had significantly more harvested lymph nodes (*P* < 0.05) and a lower overall incidence of postoperative complications (*P* < 0.05). No statistically significant differences were observed in overall survival (OS) or disease-free survival (DFS) among the 4 groups. The incidence of recurrence and death events was lower in the RATME group.

**Conclusion:**

Robot-assisted total mesoesophageal esophagectomy (RATME) could be a safe technique. Integrating mesoesophagus theory with robotic surgery achieved superior perioperative outcomes, including reduced intraoperative bleeding, increased lymph nodes dissected, lower complication rates, and accelerated recovery, and it may bring about a better long-term outcome.

Esophageal cancer ranks as the seventh leading cause of cancer death worldwide according to the 2022 Global Cancer Observatory (GLOBOCAN) report.^1^ For patients with resectable esophageal cancer, surgery remains the main treatment method.^2^ With the development of endoscopic technology and the renewal of surgical concepts, combined thoracoscopic and laparoscopic esophageal cancer resection has gradually replaced open surgery as the mainstream surgical method due to its advantages such as less intraoperative bleeding, lower incision infection rate, fewer pulmonary complications, and no impact on long-term survival rate.^3^

In recent years, the introduction of robotic surgical systems has further promoted the development of esophageal cancer surgery toward more minimally invasive and precise directions.^4^ Its flexible instrument arm, tremor-filtering function, and high-definition magnified field of view help surgeons perform precise anatomic operations, protect important structures and perhaps achieve more thorough lymph node dissection.^5,6^ The ROBOT trial has shown that robot-assisted minimally invasive esophagectomy (RAMIE) can reduce the incidence of surgery-related complications and cardiopulmonary complications, alleviate postoperative pain, promote short-term functional recovery of patients, and improve quality of life.^7,8^

At the theoretical level, the concept of membrane anatomy originated in colorectal surgery initially, suggesting that the mesentery contains nerves, blood vessels, and regional lymphatic drainage systems.^9^ It has shown that complete resection of the mesentery during rectal and colonic cancer surgeries significantly reduces tumor recurrence and improves patient prognosis.^10^

Given the similar origins of the esophagus and intestinal tract, some scholars believed in the existence of the “mesoesophagus” and pursued ongoing investigations.^11,12^ Hwang et al.^13^ confirmed that during the fifth week of fetal development, the esophagus forms a mesentery-like structure, which is sculpted by the enlarging lungs and pleural cavity. And, Sun et al.^14^ demonstrated via thoracoscopic *in vivo* observation the presence of a double-layered fascial structure originating between the descending aorta and the esophagus, containing vessels, lymphatics, and nerves. Moreover, researchers, using magnetic resonance imaging (MRI) and computed tomography (CT), have validated the presence of a membranous loose connective tissue space around the esophagus within vessels and lymph nodes,^12^ demonstrating significant pathobiologic relevance as this region correlates with prognosis in locally advanced squamous cell carcinoma.^15^ Akiyama et al.^16^ used the concept of total mesoesophageal excision (TME) for esophageal cancer and emphasized resecting the entire esophageal mesentery encompassing the tumor, regional lymph nodes, nerves, vessels, and adipose tissue. Studies confirm that TME reduces intraoperative bleeding, lowers the risk of tumor dissemination, decreases postoperative recurrence, and perhaps improves prognosis.^17,18^

Clinicians are dissatisfied with the current state of esophageal surgery. The recurrence rate after esophageal cancer surgery has decreased compared with the past, but it remains a cause for wide concern. Compared with conventional thoracoscopic esophagectomy and non-mesoesophageal esophagectomy, robot-assisted radical resection of esophageal cancer based on membrane anatomy theory seems to have advantages superimposed on clinical outcomes,^19^ but systematic and long-term research evidence currently is lacking.

This study retrospectively analyzed the clinical data of 159 patients who underwent radical resection of esophageal cancer in 2 centers from January 2019 to March 2025. They were divided into 4 groups according to the surgical methods: robot-assisted total mesoesophageal esophagectomy (RATME) group, robot-assisted minimally invasive esophagectomy invasive esophagectomy (RAMIE) group, video-assisted total mesoesophageal esophagectomy (VATME) group, and video-assisted minimally invasive esophagectomy (VAMIE) group. The analysis aimed to explore whether the use of robots and thoracoscopy under the guidance of the mesoesophagus theory can lead to better clinical efficacy and safety in radical resection of esophageal cancer.

## Methods

### Study Design

**Fig. 1:**
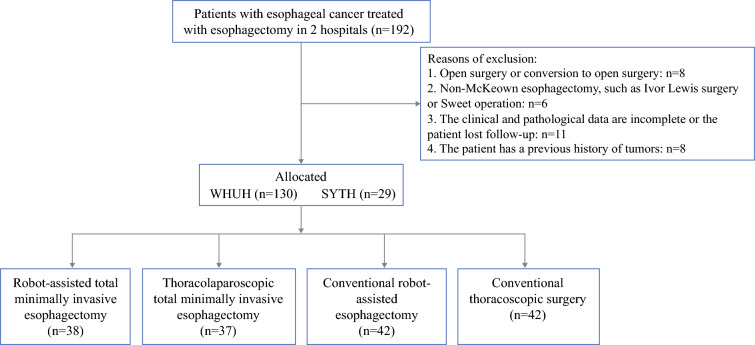
Flow diagram of data collection

This retrospective study analyzed the data of patients with esophageal cancer who underwent surgical treatment at Wuhan Union Hospital and Shiyan City Taihe Hospital in Hubei Province (Fig. 1). After approval was obtained from the ethics committee, informed consent from the patients was not required (UHCT250608). The study did not involve any additional intervention measures, and all operations were performed in accordance with relevant guidelines and norms. Esophageal cancer staging is based on the eighth edition of the tumor-node-metastasis (TNM) staging system of the Union for International Cancer Control (UICC).^20^

### Inclusion and Exclusion Criteria

The inclusion criteria specified (1) preoperative endoscopic and pathologic diagnosis of thoracic esophageal malignant tumor 20 cm from the incisors to the esophagogastric junction line (preoperative CT assessment showed no distant metastasis and no surgical contraindications); (2) age of 20 to 85 years; (3) McKeown esophagectomy successfully completed without conversion to laparotomy or change of surgical method; (4) at least lymph node dissections in the thoracic and abdominal fields completed; and (5) complete clinical and pathological data.

The exclusion criteria ruled out (1) open surgery, intraoperative transfer open or suspended; (2) non-McKeown surgical procedures (e.g., Ivor Lewis procedure); (3) previous or combined history of other non-primary esophageal malignant tumors; (4) and incomplete clinical data or loss to follow-up evaluation for any reason.

### Operation Method

All patients underwent double-lumen tracheal intubation combined with intravenous and inhalation general anesthesia. The operation was divided into 3 parts: the thoracic cavity, the abdominal cavity, and the neck. For tumors located in the upper or middle part of the thoracic esophagus, thoracic surgery should be performed first (prone position), followed by abdominal surgery (supine position). For tumors located in the lower thoracic segment, abdominal operation could be performed first.

All robotic surgeries were performed using the Da Vinci robot system Xi (Intuitive Surgical, Sunnyvale, CA, USA). In the robot-assisted surgery groups, the thoracic operation was performed via robotic thoracoscopy using 3 arms and 1 incision, with the patient in the prone position. One 12-mm trocar in the sixth intercostal space between the right axillary midline and the posterior axillary line was an observation port. Meanwhile, an incision in the same intercostal space and two 8-mm instrument-arm trocars (one on each side) served as the main operating hole. In the video-assisted groups, the thoracic operation was performed in a conventional four-hole procedure, with the patient in the prone position with a pressure of 8 mmHg carbon dioxide.

**Fig. 2:**
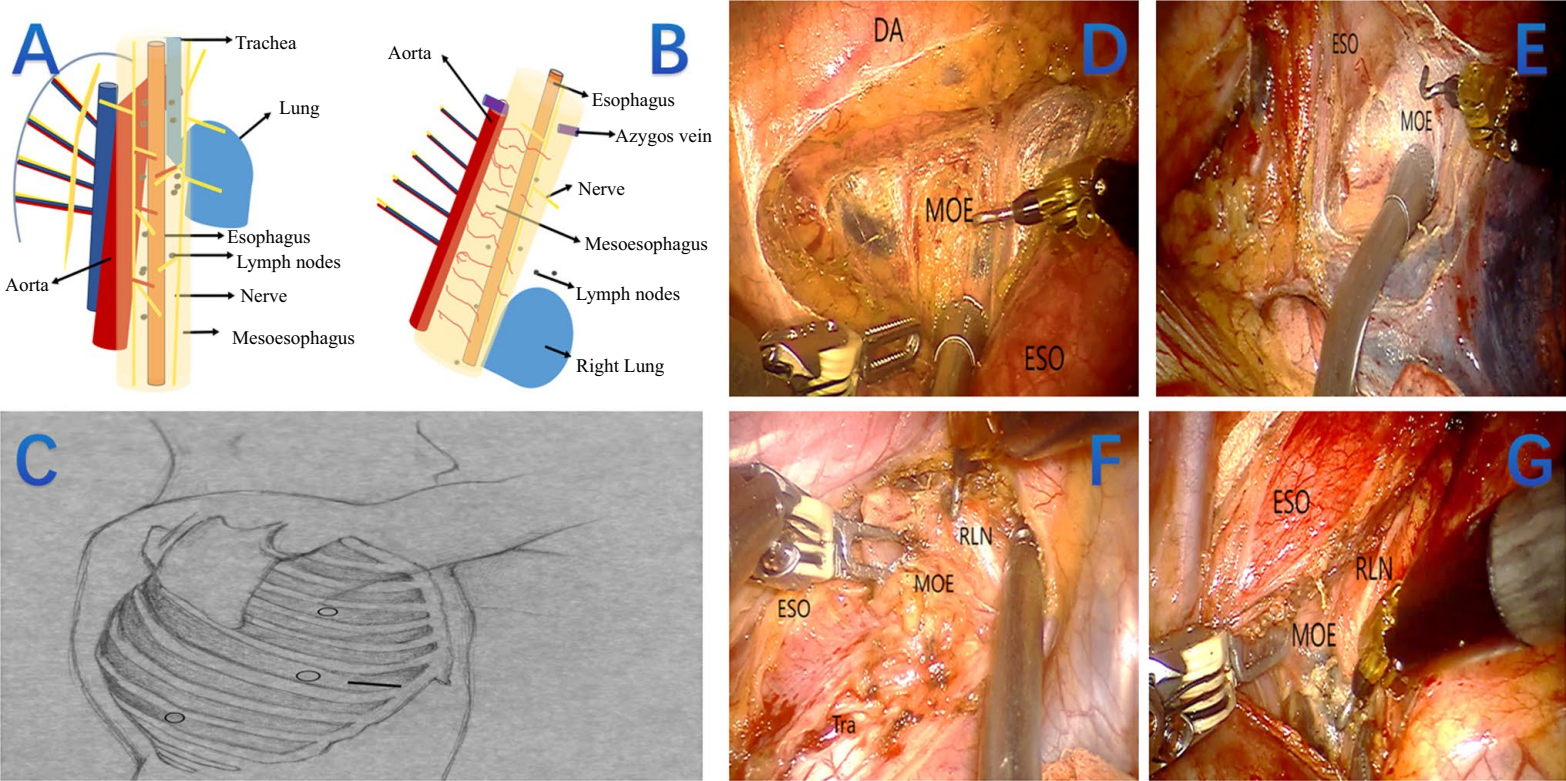
The esophagus and surrounding structures were treated as an anatomic unit. **A** The mesoesophagus in the field of anatomy (*yellow cylinder*). **B** The portion of the mesoesophagus removed during the surgical procedure (*yellow cylinder*) **C** Patient position and surgical incision (RAMIE). **D** Mesoesophagus at the spine side of the esophagus. **E** Mesoesophagus at the pericardial side. **F** Mesoesophagus along the edge of right RLN. **G** Mesoesophagus along the edge of left RLN. RAMIE, robot-assisted minimally invasive esophagectomy; DA, descending aorta; ESO, esophagus; MOE mesoesophagus. RLN, recurrent laryngeal nerve

Thoracic operation (prone position) of radical resection of esophageal cancer (TME) based on mesoesophagus theory: First, the pleura was opened longitudinally along the vertebral side of the esophagus. Beneath it, loose connective tissue was encountered, covering a fascia-like layer (Fig. 2). This tissue contained the esophageal arteries, thoracic duct, and para‑esophageal lymph nodes. Next, the pericardial side of the esophagus was opened, revealing a cavity filled with loose connective tissue that was largely avascular. Within this cavity lay the esophageal arteries, para‑esophageal lymph nodes, and subcarinal lymph nodes. This fascial layer was identified as the mesoesophagus. Thus, the esophagus, periesophageal lymph nodes, and the tissue enveloped by the mesoesophagus were removed en bloc as an intact package, while preserving the thoracic duct and azygos vein. The mesoesophagus extended bilaterally from the supracarinal esophagus to the lateral mediastinum. This region contained branches of the bronchial arteries, the recurrent laryngeal nerves, and associated lymph nodes between the carina and the thoracic inlet. Therefore, above the level of the carina, we began by opening the pleura and dissecting the right side of the mesoesophagus along the right recurrent laryngeal nerve and right subclavian artery. Subsequently, the pleura was opened up to the apex on the vertebral side, and the left side of the mesoesophagus was dissected along the course of the left recurrent laryngeal nerve. In this way, esophagectomy and lymph node dissection were accomplished synchronously via a mesoesophagus‑oriented approach, with successful preservation of the recurrent laryngeal nerves.

Conventional chest operation：When the lung tissue collapses, the azygos vein was firstly mobilized and transected, then the esophagus was mobilized from the thoracic inlet to the diaphragmatic crura. Finally, thoracic lymph nodes were dissected in groups (105,106recR, 106recL, 107, 108, 110, 111, etal).

Abdominal operation (supine position): Protect the vascular arch of the gastric mesangium, and successively dissect the left gastric mesangium vessel, the left gastric vessel, and the posterior gastric vessel, etc. According to the mesoesophagus theory, the upper part of the stomach, the left mesangial of the stomach (including nerves, blood vessels and lymphoid tissues) and the mesangial structure of the esophagus were resected as a whole. A rubber drainage tube is placed through the right operation hole in the space between the liver and stomach.

Neck operation: Make an oblique incision of about 5cm at the anterior edge of the sternocleidomastoid muscle on the left neck. Lift the esophagus and break it at the predetermined anastomosis site. The thoracic esophagus and stomach are protruded outside the abdominal cavity to create a tubular stomach. Pull the tubular stomach to the neck and perform esophagoal-tubular stomach end-to-end anastomosis. Close the incision layer by layer. Second or third field lymph node dissection is performed based on the patient’s condition and the surgeon’s judgment.

### Local and Distant Recurrence

The date when tumor recurrence was first detected through computed tomography (CT) or endoscopy during postoperative follow-up evaluation was determined as the time when the outcome event occurred. Local recurrence referred to the recurrence of the anastomotic site and regional lymph nodes. Anastomotic recurrence needed to be confirmed by gastroscopy and pathology. Superficial lymph node metastasis required pathologic confirmation by puncture biopsy. Lymph node metastases in the remaining areas were diagnosed by radiology imaging. According to TNM staging of esophageal cancer in the eighth edition of the UICC staging system, the recurrent lymph node regions were mainly divided into mediastinal, abdominal, and cervical lymph nodes. Distant metastasis was defined as non-regional metastasis (e.g., new space-occupying lesions or hypermetabolic foci in organs such as the liver and kidneys diagnosed by CT or positron emission tomography [PET]-CT, and confirmed by imaging experts).

### Parameters for Investigation

Surgical operation duration, intraoperative bleeding, total hospital stays and postoperative hospital stays, number of lymph nodes dissected, postoperative chest drainage in the first 2 days, and complications were recorded. Follow-up evaluation was performed for the effect of treatment through clinic visit records or telephone contact. Overall survival (OS) and disease-free survival (DFS) were analyzed.

### Statistical Analysis

Statistical analyses were conducted using SPSS 27.0 (IBM Corporation, Chicago, IL, USA). Categorical variables are summarized as numbers and percentages. Continuous variables are expressed as medians with ranges or as means with standard errors, as appropriate. Group differences for categorical variables were assessed using the chi-square test or Fisher’s exact test, where applicable. When significant differences were identified, multiple pairwise comparisons were performed by Bonferroni adjustment. For continuous variables, one-way analysis of variance (ANOVA) was applied, followed by the least significant difference method (LSD). Survival analysis was performed using the Kaplan-Meier method, and the survival curves were plotted using GraphPad Prism 9 (GraphPad Software, San Diego, CA, USA). The significance level was set at 0.05 (two-sided *P* values).

## Results

### Patient Characteristics

The study included 38 patients in the RATME group, 37 patients in the RAMIE group, 42 patients in the VATME group, and 42 patients in the VAMIE group. The baseline data and clinical characteristics of the four groups of patients are detailed in Table 1. Each baseline index showed no statistically significant differences among the groups (*P* > 0.05), and they were comparable.

**Table 1:** Patient characteristics^a^

	RATME (*n* = 38) *n* (%)	RAMIE (*n* = 37) *n* (%)	VATME (*n* = 42) *n* (%)	VAMIE (*n* = 42) *n* (%)	*P* Value
Gender					
Male	33 (86.84)	31 (83.78)	33 (78.57)	32 (76.19)	0.614
Female	5 (13.16)	6 (16.22)	9 (21.43)	10 (23.81)	
Mean age (years)	60.21 ± 6.58	60.49 ± 6.08	62.83 ± 6.51	62.17 ± 7.22	0.223
Mean height (cm)	166.63 ± 6.88	167.89 ± 6.17	165.64 ± 8.05	165.02 ± 7.18	0.310
Mean weight (kg)	62.62 ± 11.54	62.53 ± 7.92	60.44 ± 11.62	59.88 ± 8.58	0.442
BMI category (kg/m^2^)					0.567
<20	8 (21.05)	8 (21.62)	13 (30.95)	10 (23.81)	
20–30	28 (73.69)	29 (78.38)	28 (66.67)	32 (76.19)	
>30	2 (5.26)	0 (0.00)	1 (2.38)	0 (0.00)	
Mean albumin (g/L)	39.67 ± 3.54	39.03 ± 3.00	38.56 ± 2.95	38.31 ± 3.44	0.224
ASA-PS					0.559
1	21 (55.26)	14 (37.84)	16 (38.10)	18 (42.86)	
2	15 (39.48)	19 (51.35)	23 (54.76)	18 (42.86)	
3	2 (5.26)	4 (10.81)	3 (7.14)	6 (14.28)	
History of smoking	20 (52.63)	19 (51.35)	19 (45.24)	15 (35.71)	0.418
History of alcohol	15 (39.48)	18 (48.65)	14 (33.33)	19 (45.24)	0.526
Tumor location					0.883
Ut	4 (10.53)	2 (5.41)	4 (9.52)	6 (14.28)	
Mt	20 (52.63)	22 (59.46)	22 (52.38)	24 (57.14)	
Lt	14 (36.84)	13 (35.13)	16 (38.10)	12 (28.58)	
Mean tumor length (cm)	4.63 ± 2.61	4.53 ± 1.68	4.77 ± 2.32	5.01 ± 2.37	0.791
COPD	16 (42.11)	16 (43.24)	21 (50.00)	19 (45.24)	0.640
Diabetes	1 (2.63)	1 (2.70)	1 (2.38)	5 (11.90)	0.227
Arterial hypertension	11 (28.95))	8 (21.62)	11 (26.19)	11 (26.19)	0.909
Coronary heart disease	1 (2.63)	4 (10.81)	1 (2.38)	2 (4.76)	0.410
Cerebrovascular disease	0 (0.00)	1 (2.70)	1 (2.38)	0 (0.00)	0.605
Arrhythmology	1 (2.63)	0 (0.00)	0 (0.00)	1 (2.38)	0.860
Other chronic diseases	2 (5.26)	2 (5.41)	5 (11.90)	2 (4.76)	0.628
History of anesthesia surgery	13 (34.21)	15 (40.54)	15 (35.71)	13 (30.95)	0.846
Neoadjuvant treatment	22 (57.89)	17 (45.95)	29 (69.05)	24 (57.14)	0.235
T stage pre-therapeutic					0.680
T1	6 (15.79)	9 (24.33)	8 (19.04)	9 (21.43)	
T2	13 (34.21)	12 (32.43)	16 (38.10)	9 (21.43)	
T3	19 (50.00)	16 (43.24)	18 (42.86)	24 (57.14)	
N-stage pre-therapeutic					
N0	13 (34.21)	16 (43.24)	16 (38.10)	20 (47.62)	0.643
N+	25 (65.79)	21 (56.76)	26 (61.90)	22 (52.38)	

RATME, robot-assisted total mesoesophageal esophagectomy; RAMIE, robot-assisted minimally invasive esophagectomy invasive esophagectomy; VATME, video-assisted total mesoesophageal esophagectomy; VAMIE, video-assisted minimally invasive esophagectomy; BMI, body mass index; ASA-PS, American Society of Anesthesiologists’ Physical Status classification system. Ut, upper segment; Mt, middle segment; Lt, lower segment; COPD, chronic obstructive pulmonary disease

^a^Data are presented as mean ± standard deviation or *n* (%)

### Intraoperative Conditions and Postoperative Complications

The operation time for the RATME group was significantly longer than for the other 3 groups (*P* < 0.01; Table 2). Compared with the VAMIE group, the intraoperative blood loss in the RATME group was significantly reduced (*P* < 0.05), the thoracic drainage volume the first 2 days after the operation was significantly decreased (*P* < 0.01), and the number of thoracic lymph node dissections (P < 0.05) was significantly increased. In addition, the postoperative hospital stay in the RATME group was shorter (14.26 ± 5.78 days), but the difference between the groups was not statistically significant. Further analysis showed that the intraoperative blood loss and postoperative drainage volume in the robot groups (RATME and RAMIE) were significantly reduced compared with the thoracoscopic esophagectomy groups (VATME and VAMIE), and in the mesoesophageal esophagectomy groups (RATME and VATME) compared with the non-mesoesophageal groups (RAMIE and VAMIE).

**Table 2:** Postoperative outcomes^a^

	RATME (*n* = 38) *n* (%)	RAMIE (*n* = 37) *n* (%)	VATME (*n* = 42) *n* (%)	VAMIE (*n* = 42) *n* (%)	*P* Value
Mean surgery duration (min)	420.45 ± 88.61	354.57 ± 97.72	383.24 ± 90.72	358.57 ± 85.38	0.006^b,d^
Mean blood loss (mL)	176.32 ± 103.15	246.22 ± 158.80	221.43 ± 200.35	285.71 ± 213.07	0.048^d^
Extent of lymph node dissection					
Three fields	8 (21.05 %)	4 (10.81 %)	14 (33.33 %)	6 (14.29 %)	0.058
Two fields	30 (78.95 %)	33 (89.19 %)	28 (66.67 %)	36 (85.71 %)	
p Stage (UICC TNM 8th)					0.460
PCR	4 (10.53 %)	8 (21.62 %)	5 (11.90 %)	6 (14.29 %)	
I	12 (31.58 %)	9 (24.33 %)	16 (38.10 %)	11 (26.19 %)	
II	6 (15.79 %)	12 (32.43 %)	10 (23.81 %)	11 (26.19 %)	
III	16 (42.10 %)	8 (21.62 %)	11 (26.19 %)	14 (33.33 %)	
Histology					
Squamous cell carcinoma	37 (97.37 %)	34 (91.89 %)	42 (100 %)	40 (95.24 %)	0.239
Median no. of lymph nodes	22.95 ± 8.71	18.59 ± 7.17	22.86 ± 8.22	19.50 ± 7.73	0.028^b,e^
Thoracic nodes	13.66 ± 6.07	10.78 ± 4.49	11.36 ± 5.70	10.48 ± 5.32	0.047^b,d^
Mean total RLN nodes	2.47 ± 1.83	1.95 ± 2.08	2.24 ± 1.74	2.02 ± 1.81	0.603
Mean abdominal nodes	8.82 ± 4.84	7.73 ± 4.82	10.88 ± 5.27	8.69 ± 5.20	0.043^e^
Achievement RLN nodes rate	34 (89.47 %)	27 (72.97 %)	38 (90.48 %)	32 (76.19 %)	0.087
Mean hest drainage (ml) (first 2 days)	415.00 ± 131.31	402.84 ± 236.56	479.76 ± 220.33	562.86 ± 226.22	0.003^d,f^
Total complications	6 (15.79 %)	10 (27.03 %)	10 (23.81 %)	19 (45.24 %)	0.028^d,j^
Vocal cord paralysis	4 (10.53 %)	2 (5.41 %)	4 (9.52 %)	8 (19.04 %)	0.586
Pneumonia	3 (7.89 %)	7 (18.92 %)	5 (11.90 %)	8 (19.04 %)	0.430
Cardiac complications	1 (2.63 %)	1 (2.70 %)	4 (9.52 %)	2 (4.76 %)	0.547
Anastomotic leak	4 (10.53 %)	2 (5.41 %)	2 (4.76 %)	2 (4.76 %)	0.766
Type I (conservative)	1 (2.63 %)	2 (5.41 %)	1 (2.38 %)	0 (0.00)	
Type II (nonsurgical intervention)	3 (7.89 %)	0 (0.00)	0 (0.00)	2 (4.76 %)	
Type III (surgical intervention)	0 (0.00)	0 (0.00)	1 (2.38 %)	0 (0.00)	
Overall Infectious disease	0 (0.00)	0 (0.00)	0 (0.00)	1 (2.38 %)	>0.99
Unplanned ICU stay	0 (0.00)	0 (0.00)	1 (2.38 %)	1 (2.38 %)	>0.99
Mean postoperative hospital stay (days)	14.26 ± 5.78	14.41 ± 4.11	14.38 ± 4.36	15.12 ± 5.92	0.870
Mean total hospital stay (days)	22.08 ± 6.89	22.78 ± 5.57	21.86 ± 5.13	23.62 ± 6.35	0.540
Readmission within 30 days after discharge	0 (0.00)	0 (0.00)	1 (2.38 %)	1 (2.38 %)	>0.99
30-Day mortality	0 (0.00)	0 (0.00)	0 (0.00)	0 (0.00)	—
Hospital mortality	0 (0.00)	0 (0.00)	0 (0.00)	0 (0.00)	—

RATME, robot-assisted total mesoesophageal esophagectomy; RAMIE, robot-assisted minimally invasive esophagectomy invasive esophagectomy; VATME, video-assisted total mesoesophageal esophagectomy; VAMIE, video-assisted minimally invasive esophagectomy; UICC, Union for International Cancer Control; TNM, tumor-node-metastasis; PCR, pathologic complete remission; RLN, recurrent laryngeal nerve; ICU, intensive care unit

^a^Data are presented as mean ± standard deviation or *n* (%)

^b^Represents a significant statistical difference between RATME with RAMIE group

^c^Represents a significant statistical difference between RATME with VATME group

^d^Represents a significant statistical difference between RATME with VAMIE group

^e^Represents a significant statistical difference between RAMIE with VATME group

^f^Represents a significant statistical difference between RAMIE with VAMIE group

^j^Represents a significant statistical difference between VATME with VAMIE group

In terms of postoperative complications, one patient in the VATME group experienced respiratory failure after the operation, and one patient in the VAMIE group had poor recovery of cardiopulmonary function. Those 2 patients had to have an unplanned transfer to the ICU. After effective treatment, they recovered and were discharged. No deaths occurred during hospitalization or within 30 days after the operation.

The total complication rate was lowest in the RATME group (6 cases). In the RATME group, vocal cord paralysis occurred in four cases, pneumonia in 3 cases, arrhythmia in one case, and anastomotic leak in four cases (overlap occurred). The VAMIE group had the highest incidence of complications (19 cases). Pneumonia occurred in eight cases, vocal cord paralysis in eight cases, cardiac complication in 2 cases, and anastomotic leak in 2 cases. All complications were effectively controlled after the treatment.

### Subgroup Analysis of Patients After Neoadjuvant Therapy

The subgroups of patients receiving neoadjuvant therapy were compared (Table 3). The results were consistent with the overall analysis trend: the operation time of patients in the TME groups (RATME and VATME) was significantly longer than in the non-mesoesophageal esophagectomy groups, whereas the total lymph node dissections were more and the drainage volumes the first 2 days after the operation were significantly less than in the non-mesoesophageal esophagectomy groups. The RATME procedure led to 181.82 ± 113.96 ml of blood loss, which was statistically less than 322.92± 253.23 ml in VAMIE. The 4 groups did not differ significantly in total hospital stays, total complications, or incidence of vocal cord paralysis.

**Table 3:** Subgroup analysis of neoadjuvant therapy^m^

	RATME (*n* = 22) *n* (%)	RAMIE (*n* = 17) *n* (%)	VATME (*n* = 29) *n* (%)	VAMIE (*n* = 24) *n* (%)	*P* Value
Immunotherapy with chemotherapy	20 (90.91)	14 (82.35)	25 (86.21)	15 (62.50)	0.095
Chemoradiotherapy	2 (9.09)	2 (11.76)	1 (3.45)	1 (4.17)	
Chemotherapy	0 (0.00)	1 (5.88)	0 (0.00)	3 (12.50)	
Immunotherapy with chemoradiotherapy	0 (0.00)	0 (0.00)	3 (10.34)	4 (16.67)	
ypTNM stage					
PCR	4 (18.18)	8 (47.06)	5 (17.24)	6 (25.00)	0.117
pT0N+	0 (0.00)	0 (0.00)	1 (3.45)	1 (4.17)	>0.99
I–II	6 (27.27)	6 (35.29)	14 (48.28)	9 (37.50)	0.221
III^n^	12 (54.55)	3 (17.65)	9 (31.03)	8 (33.33)	0.109
Lymphovascular invasion (L1 status)	6 (27.27)	2 (11.76)	4 (13.79)	4 (16.67)	0.535
Perineural invasion (Pn1 status)	4 (18.18)	2 (11.76)	5 (17.24)	3 (12.50)	0.763
Tumor regression (TRG)					0.673
0	4 (18.18)	8 (47.06)	5 (17.24)	6 (25.00)	
1	6 (27.27)	4 (23.53)	7 (24.14)	7 (29.17)	
2	6 (27.27)	2 (11.76)	7 (24.14)	4 (16.67)	
3	6 (27.27)	3 (17.65)	10 (34.48)	7 (29.17)	
Mean surgery duration (min)	443.00 ± 82.98	371.12 ± 81.09	391.72 ± 100.34	362.54 ± 71.87	0.012^b,c,d^
Mean blood loss (mL)	181.82 ± 113.96	161.76 ± 96.32	237.93 ± 228.60	322.92 ± 253.23	0.037^d,f^
Mean chest drainage (ml) (first 2 days)	390.00 ± 127.28	487.06 ± 244.69	462.07 ± 173.54	609.58 ± 207.46	0.002^d,j^
Total complications	2 (9.09)	5 (29.41)	7 (24.14)	8 (33.33)	0.310
Vocal cord paralysis	2 (9.09)	2 (11.76)	2 (6.90)	5 (20.83)	0.628
Mean no. of lymph nodes	23.23 ± 9.49	20.29 ± 8.55	22.93 ± 7.85	19.25 ± 6.02	0.244
Mean total hospital stay (days)	21.00 ± 5.61	22.47 ± 6.92	21.14 ± 3.30	22.42 ± 3.74	0.614

RATME, robot-assisted total mesoesophageal esophagectomy; RAMIE, robot-assisted minimally invasive esophagectomy invasive esophagectomy; VATME, video-assisted total mesoesophageal esophagectomy; VAMIE, video-assisted minimally invasive esophagectomy; ypTNM, post-neoadjuvant pathologic tumor-node-metastasis; PCR, pathologic complete remission; pT0N+, no residual tumor cells were found at the primary site, but cancer metastasis still existed in the regional lymph nodes; TRG, tumor regression grade

^m^Data are presented as mean ± standard deviation or *n* (%)

^n^Patients with stage pT0N+ have been separately listed who are not included in this group

^b^Represents a significant statistical difference between RATME with RAMIE group

^c^Represents a significant statistical difference between RATME with VATME group

^d^Represents a significant statistical difference between RATME with VAMIE group

^e^Represents a significant statistical difference between RAMIE with VATME group

^f^Represents a significant statistical difference between RAMIE with VAMIE group

^j^Represents a significant statistical difference between VATME with VAMIE group

### Recurrence and Survival Outcomes

**Fig. 3:**
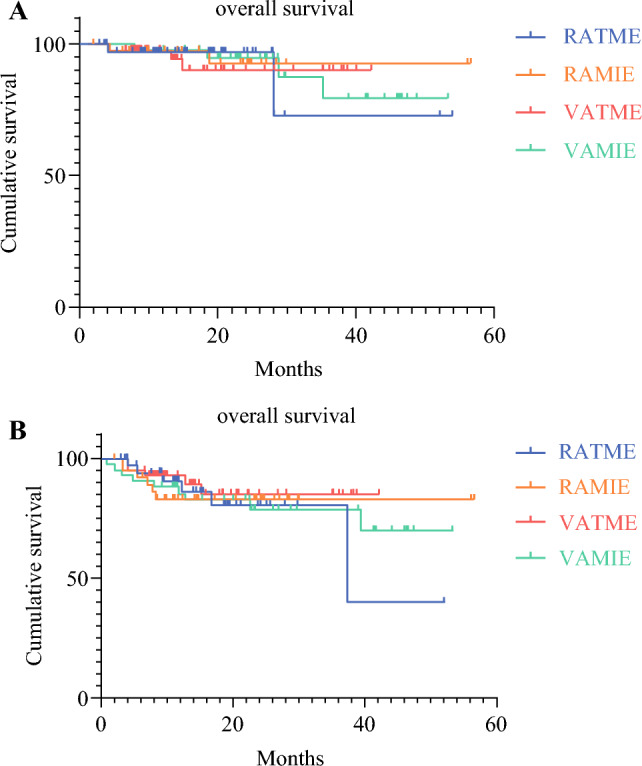
Survival analysis results: **A** 3-Year overall survival (OS) rate curves of the four groups of patients (*P* > 0.05). **B** 3-Year disease-free survival (DFS) curves of the four groups of patients (*P* > 0.05)

Follow-up evaluation was performed for 16.74 ± 11.35 months in the RATME group, for 20.27 ± 11.53 months in the RAMIE group, for 20.28 ± 11.65 months in the VATME group, and for 26.67 ± 12.26 months in the VAMIE group. The OS rates for the 4 groups were respectively 94.74 %, 94.59 %, 95.24 %, and 92.86 %, and the DFS rates were respectively 84.21 %, 83.78 %, 88.10 %, and 78.57 %. The four groups of patients had no statistically significant difference in OS or DFS (*P* > 0.05; Fig. 3). There was no significant difference in the recurrence sites of lymph nodes and the rate of distant metastasis among the 4 groups (*P* > 0.05; Table 4). One patient died of COVID-19 infection 5 months after the operation (no signs of recurrence were observed at the last reexamination) in the RATME group, whereas one patient in the VATME group died of tracheoesophageal fistula after the operation, and one patient in the VAMIE group died of anastomotic fistula complicated with infection (all deaths occurred approximately 2 months after the operation).

**Table 4:** Survival rates and recurrence sites after esophagectomy (there is some overlap in recurrence sites)

	RATME (*n* = 38) *n* (%)	RAMIE (*n* = 37) *n* (%)	VATME (*n* = 42) *n* (%)	VAMIE (*n* = 42) *n* (%)	*P* Value
Overall survival	36 (94.74)	35 (94.59)	40 (95.24)	39 (92.86)	>0.99
Disease-free survival	32 (84.21)	31 (83.78)	37 (88.10)	33 (78.57)	0.698
Overall recurrence	5 (13.16)	6 (16.22)	4 (9.52)	8 (19.05)	0.644
Liver	1 (2.63)	3 (8.11)	0 (0.00)	2 (4.76)	0239
Bone	0 (0.00)	0 (0.00)	1 (2.38)	1 (2.38)	>0.99
Kidney	1 (2.63)	0 (0.00)	0 (0.00)	1 (2.38)	0.860
Lung	1 (2.63)	1 (2.70)	3 (7.14)	2 (4.76)	0.839
Mediastinal lymph node	2 (5.26)	4 (10.81)	3 (7.14)	5 (11.90)	0.718
Abdominal lymph node	2 (5.26)	4 (10.81)	0 (0.00)	4 (9.52)	0.118
Cervical lymph node	1 (2.63)	0 (0.00)	2 (4.76)	0 (0.00)	0.509
Pleural dissemination	0 (0.00)	0 (0.00)	0 (0.00)	1 (2.38)	>0.99
Local recurrence	0 (0.00)	1 (2.70)	0 (0.00)	0 (0.00)	>0.99

RATME, robot-assisted total mesoesophageal esophagectomy; RAMIE, robot-assisted minimally invasive esophagectomy invasive esophagectomy; VATME, video-assisted total mesoesophageal esophagectomy; VAMIE, video-assisted minimally invasive esophagectomy

## Discussion

The study found that isolated cancer cells existed in the mesogastrium of resected gastric cancer specimens. This proved that the cancer cells may have infiltrated the mesogastrium, leading to the theory of the “fifth route of tumor metastasis.”^21,22^ Based on the shared developmental foundation with the intestine, the concept of the mesoesophageal was proposed later.^11,23^ Further anatomic investigations showed the mesoesophageal as a double-layered membranous connective tissue structure connecting the esophagus to adjacent structures including the aorta.^12^ And in colorectal cancer surgery, TME shares principles with complete mesogastrium excision: meticulous dissection along fascial planes while preserving membrane integrity effectively avoids vascular and lymphatic injury, reducing intra- and postoperative hemorrhage and severe seroma formation. Furthermore, this approach theoretically minimizes intraoperative tumor cell dissemination, ensures more thorough lymph node dissection, and may improve R0 resection rates, offering hope for enhanced oncologic outcomes.^14,16,17^

Moreover, in terms of hardware, robotic surgery represents a significant advancement in the field of surgery.^24^ Randomized controlled clinical trials in Asia and Europe have shown that compared with conventional minimally invasive esophagectomy (MIE), RAMIE can reduce complications of esophageal surgery and help collect more lymph nodes.^25,26^ Therefore, our study aimed to systematically compare the clinical value of TME combined with RAS (RATME) and conventional thoracoscopic non-TME surgery (VAMIE) for resectable esophageal cancer (including those receiving neoadjuvant therapy), especially to explore the safety and efficacy of their combination, an area that has not been fully clarified in previous studies.

In this study, total mesoesophageal excision (RATME, VATME) and robotic surgery were performed by experienced surgeons. However, conventional thoracoscopic non-TME surgeries (RAMIE, VAMIE) do not deliberately follow the mesangial anatomic level. The results show that surgeries based on mesoesophageal theory (especially RATME) can minimize damage to blood vessels and achieve complete removal of lymph nodes, significantly reducing unplanned blood loss and early postoperative drainage volume, and contributing to collection of more lymph nodes dissected (especially thoracic lymph nodes), which is consistent with previous reports.^14,17,27^

Among the four groups of patients receiving neoadjuvant therapy, the the number of lymph nodes did not differ significantly, although patients undergoing TME surgery had more nodes than the non-TME group. This may have been related to lymphoid tissue fusion induced by chemotherapy drugs or radiation, which can make lymph nodes difficult to harvest or prone to fragmentation. On the other hand, the TME groups experienced less bleeding and fewer postoperative complications, particularly vocal cord paralysis, underscoring the significant clinical value of the TME concept in complex esophageal surgery.

Regarding long-term survival rates, we present the first more comprehensive comparative evidence with relatively extended follow-up periods. The TME group demonstrated fewer recurrence and mortality events, together with a lower mediastinal recurrence rate. However, no statistically significant differences in OS or DFS were observed among the four groups. Although some previous studies suggested that the TME principle may be associated with survival benefits,^16^ our data in this study could not confirm this advantage. Given the limitations of the study, we recommend interpreting these results with caution. In other words, the lack of significant differences may stem from the relatively limited sample size and short follow-up periods, potentially preventing the study from achieving sufficient statistical power to detect clinically relevant differences in survival outcomes. Nevertheless, combined with the significantly higher lymph node retrieval rate, the observed numeric trend supports a cautious hypothesis: total mid-esophageal resection may reduce the risk of local recurrence and improve long-term survival by comprehensively clearing tumor tissue and its associated lymphatic drainage.^19^ Therefore, we are currently conducting a larger cohort study and calling for further validation with more participants and a longer follow-up period.

Conventional thoracoscopic esophagectomy has limitations when deep and fine dissection is performed in a narrow space, which can easily lead to deviations at the operational level, increased bleeding, and damage to adjacent structures. The results of this study confirm this point: even for the same type of surgery (mesoesophageal esophagectomy or non-mesoesophageal esophagectomy), the intraoperative blood loss and postoperative drainage volume in the thoracoscopic surgery groups (VATME and VAMIE) were higher than those in the corresponding robotic groups (RATME and RAMIE), and the complication rate was the highest in the VAMIE group. However, thanks to the surgeons’ proficient mastery of thoracoscopic surgery, the operation time of the thoracoscopic surgery group was relatively shorter. It is worth noting that the tissue adhesions and blurred anatomic layers caused by neoadjuvant therapy significantly increase the difficulty of the surgery.^28,29^ In this case, the advantages of the robot system in fine anatomic separation are more prominent, which is also reflected in the subgroup analysis of this study (the robot group could still maintain a low bleeding trend in complex situations).

This study had several limitations that warrant clarification. First, as a retrospective analysis, it may have been subject to potential selection bias. Second, although the sample size was substantial for a single-center study of such complex procedures, the relatively limited sample size and follow-up duration may have resulted in insufficient statistical power to detect significant differences in long-term survival outcomes such as OS and DFS. This limitation may constrain the conclusions drawn from the available evidence. Third, although we used standard multiple comparison methods, post hoc pairwise comparisons require more context-specific interpretation based on clinical relevance. Future studies should address these limitations, particularly by using larger sample sizes and extended follow-up periods to enhance the reliability and applicability of the current findings.

In conclusion, this study showed that the mesoesophagus theory and robotic surgery are beneficial for radical esophagectomy. Using surgical robots and following the principles of “total mesoesophageal excision” helps increase the number of lymph node dissections, reduce intraoperative damage and bleeding, and promote rapid postoperative recovery of patients. This explains that in some clinical indicators, surgeries based on mesoesophagus theory have better results than non-mesoesophageal esophagectomy, and robot-assisted surgeries have better results than thoracoscopic esophagectomy. Importantly, the combined surgical approach does achieve more excellent clinical outcomes. The exact longer-term survival value awaits further confirmation by high-quality prospective randomized controlled trials.
